# Supplementary material for: The γ-Tubulin Ring Complex promotes mitotic spindle integrity and acts as a microtubule minus-end cap during mitosis
Source: bioRxiv. 2026 Apr 11:2026.04.10.717779. Preprint. [Version 1] doi: 10.64898/2026.04.10.717779 (PMC13082017; doi:10.64898/2026.04.10.717779)
Supplement: Supplement 1 [file NIHPP2026.04.10.717779v1-supplement-1.pdf]

bioRxiv preprint doi: https://doi.org/10.64898/2026.04.10.717779; this version posted April 11, 2026. The copyright holder for this preprint (which was not certified by peer review) is the author/funder. This article is a US Government work. It is not subject to copyright under 17 USC 105 and is also made available for use under a CC0 license.

Legends for supplementary figures

Supplementary Figure 1: Strategy for tagging individual γ-TuRC subunits and validation of cell lines

A- Schematic for C-terminus tagging of the endogenous *GCP2* locus in DLD-1 cells. GCP2 was tagged with a NG fluorescent tag for visualization and 3X mini-AID tag to mediate targeted depletion. Hygromycin used as a selection marker for clone production was cleaved off by an adjacent P2A site. Primers used for confirming biallelic integration were designed and targeted outside the left and right homology arms as indicated.

B- Genomic PCR confirming the biallelic tagging of GCP2 in DLD-1 cells. Control cells (DLD-1) show only the untagged allele (1978 bps) and the GCP2 $^{NG:AID}$ cells show only the tagged allele (4492 bp, marked by the asterisk).

C- Schematic for N-terminus tagging at the endogenous *GCP4* locus with a NG fluorescent tag, a 3X mini-AID tag and a hygromycin selection marker. Primers designed and targeted outside the homology arms were used for confirming the biallelic integration at the GCP4 genomic locus.

D- Genomic PCR confirming the biallelic tagging at the *GCP4* locus. Control cells (DLD-1) show only the untagged allele (2162 bps) and the $^{AID:NG}$ GCP4 cells show only the tagged allele (4625 bp, marked by the asterisk). The clone #17-3 highlighted in red was used in this manuscript.

E- Schematic for N-terminus tagging at the endogenous *GCP6* locus with a NG fluorescent tag, a 3X mini-AID tag and a hygromycin selection marker. Primers used for confirming integration were designed and targeted as indicated.

F- Genomic PCR confirming the biallelic tagging of GCP6 in DLD-1 cells. (left) The GCP6_gPCR_F_OUT and GCP6_gPCR_R_IN set showed a control band of 1513 bp and 4018 bp band indicating targeted insertion, (right) The GCP6_gPCR_F_IN and GCP6_gPCR_R_OUT combination showed a control band of 1661 bp and 4166 bp band indicating targeted insertion at the *GCP6* locus.

Supplementary Figure 2: Co-dependence of γ-TuRC subunits for centrosomal targeting

A- Schematic for the protocol used for Western Blotting experiments in B and C.

bioRxiv preprint doi: https://doi.org/10.64898/2026.04.10.717779; this version posted April 11, 2026. The copyright holder for this preprint (which was not certified by peer review) is the author/funder. This article is a US Government work. It is not subject to copyright under 17 USC 105 and is also made available for use under a CC0 license.

B, C- Western blot indicating persistence of γ-TuRC subunits upon depletion of GCP4 and GCP6 for various time points as indicated. DLD-1 control is depicted in lane 1. β-actin was used as a loading control.

D- Schematic for the protocol for Immunofluorescence analysis in E and F.

E, F- Immunofluorescence staining for γ-TuRC subunits (orange) upon GCP4 and GCP6 depletion as shown. Centrosomes (red) were marked by PCNT or NuMA as indicated in the diagrams. DNA was visualized with Hoechst. Scale bars : 10 µm.

Supplementary Figure 3: Spindle collapse upon GCP2 depletion visualized by endogenous tubulin tagging

(top) - Schematic for protocol for live imaging experiments to visualize MTs. (bottom) Mitotic progression of GCP2 $^{NG:AID}$ cells simultaneously tagged with ^{mCherry} α-tubulin. Auxin was added for 3h prior to imaging to mediate GCP2 depletion. RCC1 ^{IFP} was used to visualize chromatin.

Supplementary Figure 4: Persistence of KT - MT attachment after spindle collapse induced by loss of γ-TuRC

(top) - Schematic for protocol followed for Immunofluorescence experiment under cold shock for visualizing stable MTs. (bottom)- visualization of cold- stable MTs using α- tubulin (red) upon loss of γ-TuRC subunits as indicated. CREST was used as the KT marker. DNA was visualized with Hoechst. Merged images represent the γ-TuRC subunit indicated (green), α- tubulin (red) and CREST (grey). Images shown are single z sections. Scale bars : 10 µm

Supplementary Figure 5: Depletion of γ-TuRC subunits at metaphase results in a weaker collapsed spindle

A- (top) Schematic showing protocol used for live imaging. A cell captured at metaphase was chosen and auxin was added immediately before imaging. (bottom) Mitotic progression of GCP2$^{NG:AID}$ cells simultaneously tagged with ^{mCherry} Eg5 following auxin addition. RCC1 ^{IFP} was used to visualize chromatin.

B- (top) Schematic showing protocol used for immunofluorescence staining. ProTAME was used to arrest the cells in prometaphase. Auxin was added 3h prior to fixation of cells to deplete the

bioRxiv preprint doi: https://doi.org/10.64898/2026.04.10.717779; this version posted April 11, 2026. The copyright holder for this preprint (which was not certified by peer review) is the author/funder. This article is a US Government work. It is not subject to copyright under 17 USC 105 and is also made available for use under a CC0 license.

γ-TuRC subunits as indicated. (bottom) Immunofluorescence staining of metaphase arrested cells for α-tubulin (red). γ-TuRC (green) and PCNT (grey) which was used as the centrosome marker are shown. DNA was visualized with Hoechst. Scale bars : 10 μm

Supplementary Figure 6: γ-TuRC subunits show slow turnover rate at centrosomes

A, B- FRAP experiment showing GCP4 or GCP6 turnover dynamics. The region at centrosomes targeted for bleaching is indicated by the solid red circle in the prebleach images. Images indicating the region immediately after bleaching and over a course of 14 min are shown. Scale bars : 10 μm.

Supplementary Figure 7: Verification of siRNA mediated KIF2A knockdown

(top) Schematic showing the protocol used for western blots following siRNA mediated depletion. (bottom) Western blot showing confirmation for KIF2A downregulation using siRNA at different timepoints (24h, 48h, 72h, 96h). KIF2A levels are indicated. β- actin was used as a loading control.

bioRxiv preprint doi: https://doi.org/10.64898/2026.04.10.717779; this version posted April 11, 2026. The copyright holder for this preprint (which was not certified by peer review) is the author/funder. This article is a US Government work. It is not subject to copyright under 17 USC 105 and is also made available for use under a CC0 license.

Supplementary Table1: Primers used for the constructing the guide and donor plasmids used in this study.

Primer#	Primer Name	Sequence
1	LHA_GCP2_C-term_F	ggaggcggtagtggcggtggtggatccgtgctagcGTGTGACATTCTGTGCAACTGGGCTG
2	LHA_GCP2_C-term_R	gccaccacctccggaTGCTTGAGCTGTCACAGCCACCCGAGGTGCTGGCGCGGGAGGGCCCCTGAGCACGGGGACTTGGGGGGTGGCCTTCTGGC
3	GCP2_NG_P2A_Hygro_F	gtgacagctcaagcaTCCGGAGGTGGTGGCAGCGGCGGAG
4	GCP2_NG_P2A_Hygro_R	tgtcacagccagggcCTATTCCTTTGCCCTCGGACGAGTGCTGGGGC
5	RHA_GCP2_C-term_F	agggcaaaggaatagGCCCTGGCTGTGACAGGAAGGAAGG
6	RHA_GCP2_C-term_R	gtggtatggctgattatgatctagagtcgcggccgcCCAGAATGTAACCAGAGCAGACCAACCTG
7	GCP2_gRNA1_F	caccgTGCGGTGACTGCGACCCT
8	GCP2_gRNA1_R	aaacAGGGTCGCAGTCACCGCAc
9	GCP2_gRNA2_F	caccgCAAGTGCCTGTCCTGCGG
10	GCP2_gRNA2_R	aaacCCGCAGGACAGGCACTTGc
11	LHA_GCP4_N-term_F	ggaggcggtagtggcggtggtggatccgtgctagcCACGTCCAGAATCTCGGGTCTTTGT
12	LHA_GCP4_N-term_R	tttcatgtttaaaccCAAGAGCAGTTCGTGGATCATTCCC
13	GCP4_NG_P2A_Hygro_F	cacgaactgctcttgGGTTTAAACATGAAAAAGCCTGAACTCACCGCGACGTCTGTCGAGAAGTTTCTGATCGAAAAGTTCGAC
14	GCP4_NG_P2A_Hygro_R	cacgaactgctcttgGGTTTAAACATGAAAAAGCCTGAACTCACCGCGACGTCTGTCGAGAAGTTTCTGATCGAAAAGTTCGAC

bioRxiv preprint doi: https://doi.org/10.64898/2026.04.10.717779; this version posted April 11, 2026. The copyright holder for this preprint (which was not certified by peer review) is the author/funder. This article is a US Government work. It is not subject to copyright under 17 USC 105 and is also made available for use under a CC0 license.

15	RHA_GCP4_N-term_F	ggtagtggcggtggcATGATCCACGAACTGCTCTTGGCCCtctccggctatccaggcagcatctttacatggaataaacggagtggcctgcaggtactg
16	RHA_GCP4_N-term_R	gtggtatggctgattatgatctagagtcgcggccgcTGCCACTACTAGGACTGGCCAGCAT
17	GCP4_gRNA2_F	caccGCTCTGAGCGGGTACCCT
18	GCP4_gRNA2_R	aaacAGGGTACCCGCTCAGAGC
19	GCP4_gRNA6_F	caccGCTTGTTCCAGGTGAAAA
20	GCP4_gRNA6_R	aaacTTTTCACCTGGAACAAGC
21	LHA_GCP6_N-term_F	cggtagtggcggtggtggatccgtgctagcGCCTCACCAACTTCACGCGC
22	LHA_GCP6_N-term_R	tcatgtttaaaccCAGGAGGGCCTCACACAGGT
23	GCP6_NG_P2A_Hygro_F	tgaggccctcctgGGTTTAAACATGAAAAAGCCTGAACTCACCGCG
24	GCP6_NG_P2A_Hygro_R	tgatgctggccatGCCACCGCCACTACCGCCTC
25	RHA_GCP6_N-term_F	tagtggcggtggcATGGCCAGCATCACGCAGCTgttcgacgacctgtgtgaggccctcctgcctgccgctaaaacacatctcgggcaaagaagcgtcaatagaaagagggcaaagcggagcctc
26	RHA_GCP6_N-term_R	atggctgattatgatctagagtcgcggccgcCTTAATAGCCAGCCCAGAGAGGTC
27	GCP6_gRNA8_F	caccGGCCAGCGCAGTGTGAAC
28	GCP6_gRNA8_R	aaacGTTCACACTGCGCTGGCC
29	GCP6_gRNA 19_F	caccgCAGGTGAGTCTTGGCAGC
30	GCP6_gRNA 19_R	aaacGCTGCCAAGACTCACCTGc
31	LHA_GCP5_Nterm_R	gtcatcatatgctccgcTGGCCCGTGCCGCGCCATGTTCCGC
32	GCP5_mCherry_P2A_F	gcgcggcacgggccagcGGAGCATATGATGACCGAGTACAAGCCCACGG

bioRxiv preprint doi: https://doi.org/10.64898/2026.04.10.717779; this version posted April 11, 2026. The copyright holder for this preprint (which was not certified by peer review) is the author/funder. This article is a US Government work. It is not subject to copyright under 17 USC 105 and is also made available for use under a CC0 license.

33	GCP5_mCherry_P2A_R	ccgtgccgcgccatgcCTCCGAATTCACCTCCGCCGCCGGC
34	RHA_GCP5_Nterm_F	gaggtgaattcggaggcATGGCGCGGCACGGGCCACCGTGGA
35	LHA_GCP5_Nterm_F	ggaggcggtagtggcggtggtggatccgtgctagcACAGCACCTCTGGCCAGGTGGGATTATGATACCCCC
36	RHA_GCP5_Nterm_R	gtggtatggctgattatgatctagagtcgcggccgcATTAGGTGACTCAGCGTGCTACGTGCCAGGAGCTGTGCAG
37	GCP5_gRNA1_F	caccgCGCGTCCAACCGACTCCA
38	GCP5_gRNA1_R	aaacTGGAGTCGGTTGGACGCGc
39	GCP5_gRNA6_F	caccGTCGGTTGGACGCGCAGC
40	GCP5_gRNA6_R	aaacGCTGCGCGTCCAACCGAC
41	RHA_CDK5RAP2_Nterm_R	atggctgattatgatctagagtcgcggccgcGCACTGATTATGTGGCAGGC
42	LHA_CDK5RAP2_Nterm_R	gtcatcatatgctccgcTTCCAACACCAAGTCCATCATG
43	CDK5RAP2_Puro_P2A_mCherry_F	cttggtgttggaagcGGAGCATATGATGACCGAGTACAAG
44	CDK5RAP2_Puro_P2A_mCherry_R	cttggtgttggaagcGGAGCATATGATGACCGAGTACAAG
45	LHA_CDK5RAP2_Nterm_F	cggtagtggcggtggtggatccgtgctagcCTGGCCCTCAGTTTTCTGAC
46	RHA_CDK5RAP2_Nterm_F	ggtgaattcggaggcATGATGGACTTGGTGTTGGAAG
47	CDK5RAP2_gRNA2_F	caccGAGGACGTCACCGTCCCT
48	CDK5RAP2_gRNA2_R	aaacAGGGACGGTGACGTCCTC
49	CDK5RAP2_gRNA3_F	caccgCGTCCCTGGGACGCTCAG

bioRxiv preprint doi: https://doi.org/10.64898/2026.04.10.717779; this version posted April 11, 2026. The copyright holder for this preprint (which was not certified by peer review) is the author/funder. This article is a US Government work. It is not subject to copyright under 17 USC 105 and is also made available for use under a CC0 license.

50	CDK5RAP2_gRNA3_R	aaacCTGAGCGTCCCAGGGACGc
51	LHA_NuMA_Nterm_F	gtggcggtggtggatccgtgctagcAATCCCCCATTTGAACTGTG
52	LHA_NuMA_Nterm_R	tatgctccagGAGTGTCATCTTGGTGATGC
53	NuMA_Puro_P2A_mCherry_F	gatgacactcCTGGAGCATATGATGACCGAGTAC
54	NuMA_Puro_P2A_mCherry_R	ggagtgtcatGCCTCCGAATTCACCTCCGC
55	RHA_NuMA_Nterm_F	attcggaggcATGACACTCCATGCTACAAGaggcgcagctctcctctcttgggtaagtagtc
56	RHA_NuMA_Nterm_R	tgattatgatctagagtcgcggccgcTAGAAAATGTATGAATGATCTAGC
57	NuMA _gRNA1_F	caccGACACTCCACGCCACCCG
58	NuMA _gRNA1_R	aaacCGGGTGGCGTGGAGTGTC
59	NuMA _gRNA8_F	caccGAGAGGAGTGCAGCCCCC
60	NuMA_gRNA8_R	tcgcaaagctTCCGGAGGTGGTGGCAGCGG
61	TubA3 _gRNA1_F	caccgTGCACTCACGCTGTGGGG
62	TubA3 _gRNA1_R	aaacCCCCACAGCGTGAGTGCAc
63	TubA3 _gRNA8_F	caccgAGATGCACTCACGCTGTG
64	TubA3 _gRNA8_R	aaacCACAGCGTGAGTGCATCTc

bioRxiv preprint doi: https://doi.org/10.64898/2026.04.10.717779; this version posted April 11, 2026. The copyright holder for this preprint (which was not certified by peer review) is the author/funder. This article is a US Government work. It is not subject to copyright under 17 USC 105 and is also made available for use under a CC0 license.

Supplementary Table2: Genomic PCR primers used for the verification of integration in the correct genomic locus

Primer#	Primer Name	Sequence
1	GCP2_gPCR_F	TGGTCATATGCCACTCCC
2	GCP2_gPCR_R	AAGGAGGAAGCGGTTTGT
3	GCP4_gPCR_F	TAAAGACAAGTCACCATCCTG
4	GCP4_gPCR_R	GTCTTCAAAGACCTACAGGTG
5	GCP6_gPCR_F_OUT	GTGGAAGATGTTGCAGGTGA
6	GCP6_gPCR_R_IN	CTCCACAAGCTCCTCCAAAC
7	GCP6_gPCR_F_IN	CTGAGTAGAGCAACCGCAAG
8	GCP6_gPCR_R_OUT	ACAATGCAGCCACCAGACTA

Antibody name	Manufacturer	Species	Catalog #,
GCP2 (anti-TUBGCP2)	Novus Biologicals	Mouse monoclonal	NBP2-21793
GCP2 (anti-TUBGCP2)	abcam	Mouse monoclonal	ab140225
GCP3 (C-3)	Santa Cruz biotechnology	Mouse monoclonal	sc-373758, Lot # B0414
GCP4 (D-5)	Santa Cruz biotechnology	Mouse monoclonal	sc-271876, Lot # G0920
GCP4 (anti-GCP4)	Novus	Rabbit polyclonal	NBP2-16628, Lot #40289
GCP5 (E-1)	Santa Cruz biotechnology	Mouse monoclonal	sc-365837, Lot # C0723
GCP6 (H-9)	Santa Cruz biotechnology	Mouse monoclonal	sc-2374063, Lot #L2624
GCP6 (anti-TUBGCP6)	Novus	Rabbit polyclonal	NBP3-30520, Lot #1
KIF2A (anti-KIF2A)	Bethyl	Rabbit polyclonal	A300-914A
α-Tubulin (anti-α-Tubulin)	Millipore	Mouse monoclonal	T6199
β-Actin (beta-Actin (13E5))	Cell signalling Technology	Rabbit monoclonal	4970L
NEDD1 (H-3)	Santa Cruz biotechnology	Mouse monoclonal	sc-398733 Lot#C0624
Pericentrin	abcam	Rabbit polyclonal	AB4448
γ-Tubulin (gamma-tubulin)	abcam	Rabbit polyclonal	AB11317
NuMA (F-11)	Santa Cruz biotechnology	Mouse monoclonal	sc-365532, Lot #C2211

bioRxiv preprint doi: https://doi.org/10.64898/2026.04.10.717779; this version posted April 11, 2026. The copyright holder for this preprint (which was not certified by peer review) is the author/funder. This article is a US Government work. It is not subject to copyright under 17 USC 105 and is also made available for use under a CC0 license.

NIH Intramural Research Program and Employee Publishing Agreement & Manuscript Cover Sheet

By signing this Cover Sheet, the Author, on behalf of NIH, agrees to the provisions set out below, which modify and supersede, solely with respect to NIH, any conflicting provisions that are in the Publisher's standard copyright agreement (the "Publisher's Agreement"). If a Publisher's Agreement is attached, execution of this Cover Sheet constitutes an execution of the Publisher's Agreement subject to the provisions and conditions of this Cover Sheet.

1. **Indemnification.** No Indemnification or "hold harmless" obligation is provided by either party.

2. **Governing Law.** This agreement will be governed by the law of the court in which a claim is brought.

3. **Copyright.** Author's contribution to the Work was done as part of the Author's official duties as an NIH employee and is a Work of the United States Government. Therefore, copyright may not be established in the United States. 17 U.S.C. § 105. If Publisher intends to disseminate the Work outside of the U.S., Publisher may secure copyright to the extent authorized under the domestic laws of the relevant country, subject to a paid-up, nonexclusive, irrevocable worldwide license to the United States in such copyrighted work to reproduce, prepare derivative works, distribute copies to the public and perform publicly and display publicly the work, and to permit others to do so.

4. **No Compensation.** No royalty income or other compensation may be accepted for work done as part of official duties. The author may accept for the agency a limited number of reprints or copies of the publication.

5. **NIH Representations.** NIH represents to the Publisher that the Author is the sole author of the Author's contribution to the Work and that NIH is the owner of the rights that are the subject of this agreement; that the Work is an original work and has not previously been published in any form anywhere in the world; that to the best of NIH's knowledge the Work is not a violation of any existing copyright, moral right, database right, or of any right of privacy or other intellectual property, personal, proprietary or statutory right; that where the Author is responsible for obtaining permissions or assisting the Publishers in obtaining permissions for the use of third party material, all relevant permissions and information have been secured; and that the Work contains nothing misleading, obscene, libelous or defamatory or otherwise unlawful. NIH agrees to reasonable instructions or requirements regarding submission procedures or author communications, and reasonable ethics or conflict of interest disclosure requirements unless they conflict with the provisions of this Cover Sheet. Notwithstanding the foregoing, the Author may, consistent with NIH policy, submit a copy of the original work to a public repository.

6. **Disclaimer.** NIH and the Author expressly disclaim any obligation in Publisher's Agreement that is not consistent with the Author's official duties or the NIH mission, described at https://www.nih.gov/about-nih. NIH and the Author do not disclaim obligations to comply with a Publisher's conflict of interest policy so long as, and to the extent that, such policy is consistent with NIH's own conflict of interest policies.

7. **For Peer-Reviewed Papers to be Submitted to PubMed Central.** The Author is a U.S. government employee who must comply with the NIH Public Access Policy, and the Author or NIH will deposit, or have deposited, in NIH's PubMed Central archive, an electronic version of the final, peer-reviewed manuscript upon acceptance for publication, to be made publicly available upon the Official Date of Publication, defined in the NIH Public Access Policy as the date on which the Final Published Article is first made available in final, edited form, whether in print or electronic (i.e., online) format. The Author and NIH agree (notwithstanding Paragraph 3 above) to follow the manuscript deposition procedures of the publisher so long as they are consistent with the NIH Public Access Policy, including making the manuscript publicly available without embargo in PubMed Central.

bioRxiv preprint doi: https://doi.org/10.64898/2026.04.10.717779; this version posted April 11, 2026. The copyright holder for this preprint (which was not certified by peer review) is the author/funder. This article is a US Government work. It is not subject to copyright under 17 USC 105 and is also made available for use under a CC0 license.

8. **Modifications.** PubMed Central may tag and modify the work consistent with its customary practices and within the meaning and integrity of the underlying work.

The NIH Deputy Director for Intramural Research approves this publishing agreement for NIH staff from the NIH Intramural Program. NIH Institute and Center Directors approve this publishing agreement for NIH staff from their respective Institutes and Centers outside the NIH Intramural Program. The NIH Principal Deputy Director approves this publishing agreement for NIH staff in the Office of the Director. A single, signed copy of this text is maintained for all works published by NIH employees, and contractors and trainees who are working at the NIH. No additional signatures beyond that of the Author are needed.

Author's name: Reham Aljumaah, Elizabeth A Turcotte, Sanjana Sundararajan, Vasilisa Aksenova, Alexei Arnaoutov and Mary Dasso

Author's Institute, Center, or OD Office: NICHD

Check if from the NIH Intramural Research Program ☑

Check if Publisher Agreement is attached ☐

Title of Manuscript/Work: The γ-Tubulin Ring Complex functions promotes mitotic spindle integrity and acts as a microtubule minus-end cap during mitosis

Name of publication: Review Commons (https://www.reviewcommons.org/)

____
Author's signature

April 9, 2026
Date

NIH OGC May 2025

bioRxiv preprint doi: https://doi.org/10.64898/2026.04.10.717779; this version posted April 11, 2026. The copyright holder for this preprint (which was not certified by peer review) is the author/funder. This article is a US Government work. It is not subject to copyright under 17 USC 105 and is also made available for use under a CC0 license.

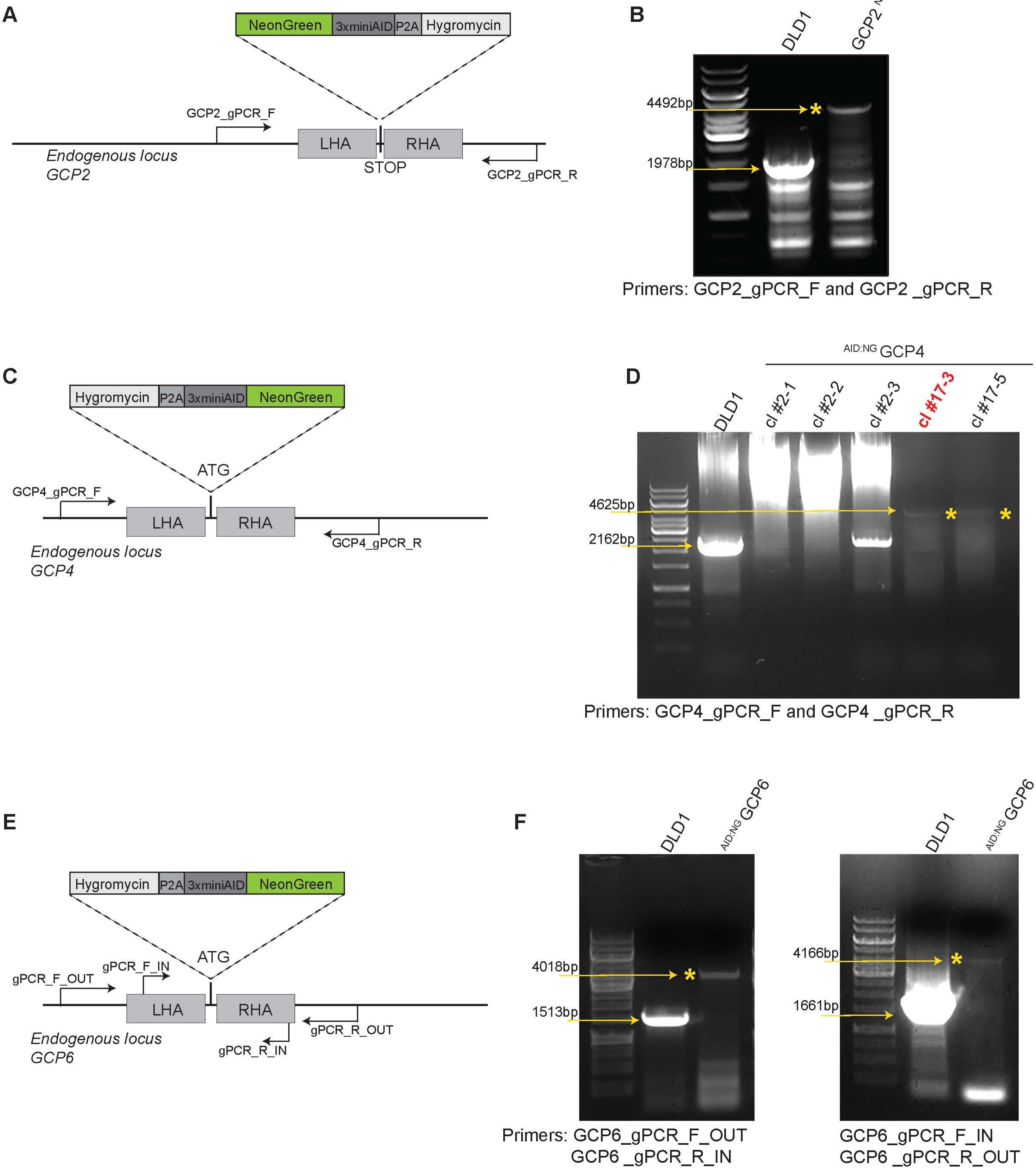

Supplementary Figure 1

bioRxiv preprint doi: https://doi.org/10.64898/2026.04.10.717779; this version posted April 11, 2026. The copyright holder for this preprint (which was not certified by peer review) is the author/funder. This article is a US Government work. It is not subject to copyright under 17 USC 105 and is also made available for use under a CC0 license.

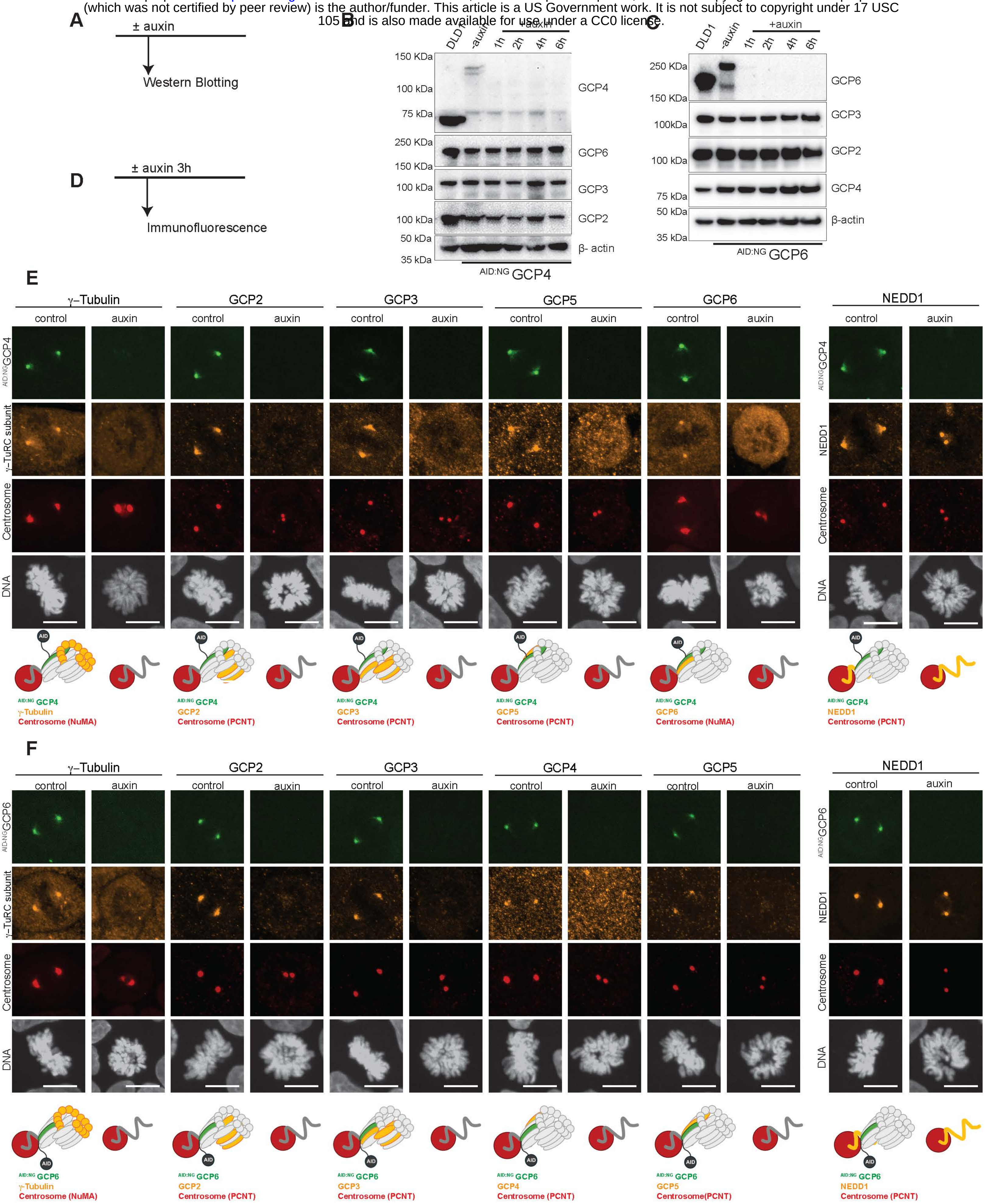

Supplementary Figure 2

bioRxiv preprint doi: https://doi.org/10.64898/2026.04.10.717779; this version posted April 11, 2026. The copyright holder for this preprint (which was not certified by peer review) is the author/funder. This article is a US Government work. It is not subject to copyright under 17 USC 105 and is also made available for use under a CC0 license.

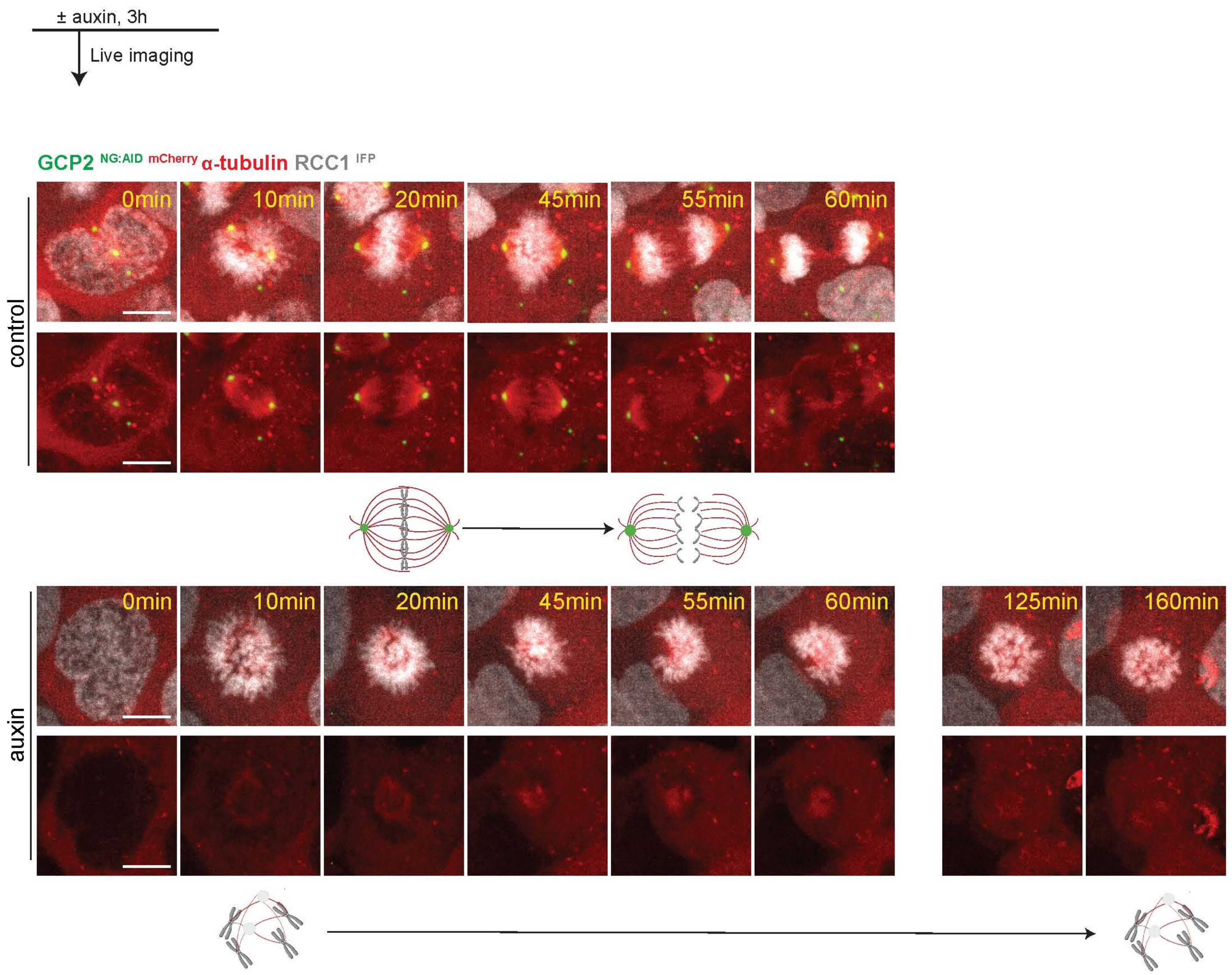

Supplementary Figure 3

bioRxiv preprint doi: https://doi.org/10.64898/2026.04.10.717779; this version posted April 11, 2026. The copyright holder for this preprint (which was not certified by peer review) is the author/funder. This article is a US Government work. It is not subject to copyright under 17 USC 105 and is also made available for use under a CC0 license.

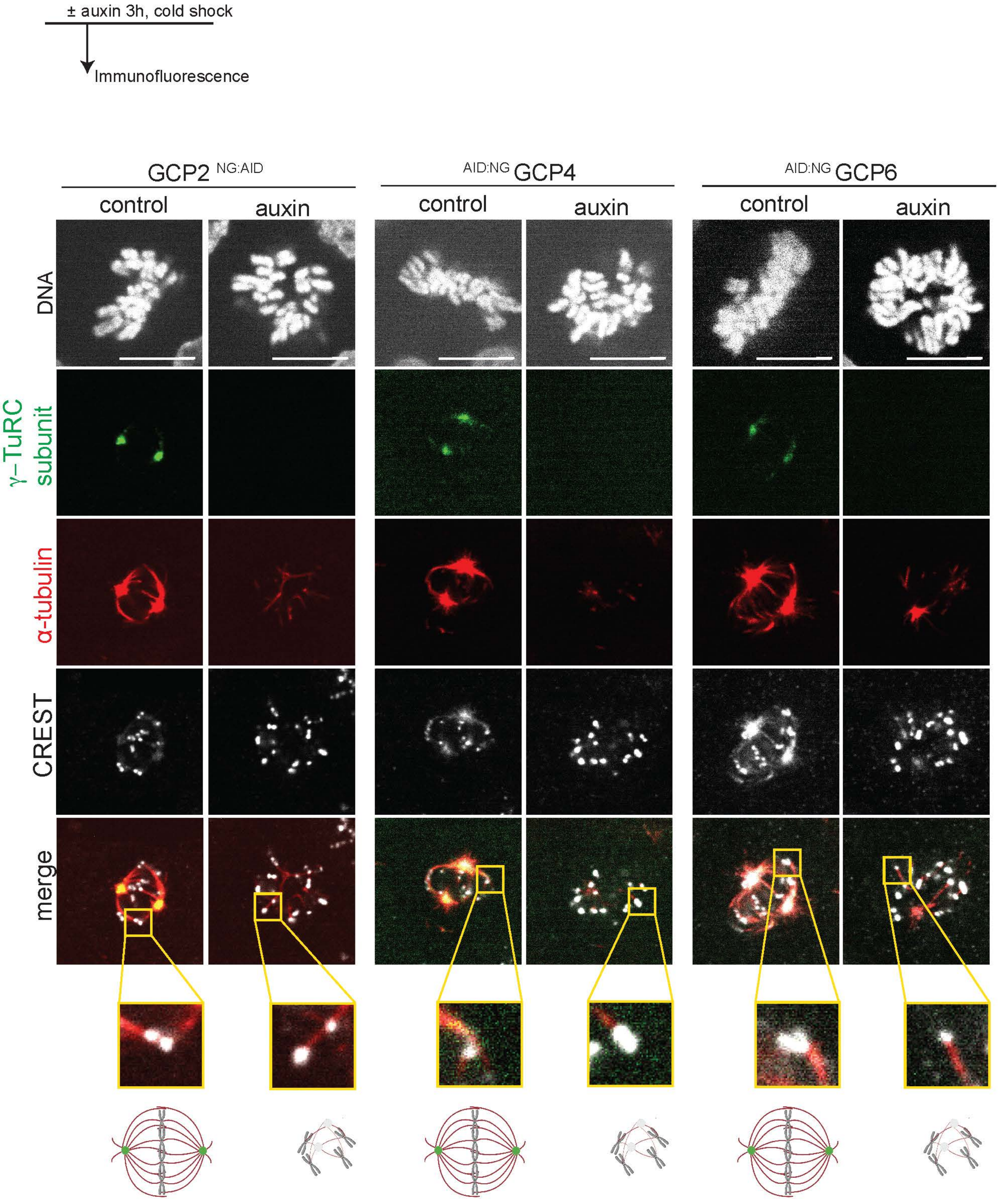

Supplementary Figure 4

bioRxiv preprint doi: https://doi.org/10.64898/2026.04.10.717779; this version posted April 11, 2026. The copyright holder for this preprint (which was not certified by peer review) is the author/funder. This article is a US Government work. It is not subject to copyright under 17 USC 105 and is also made available for use under a CC0 license.

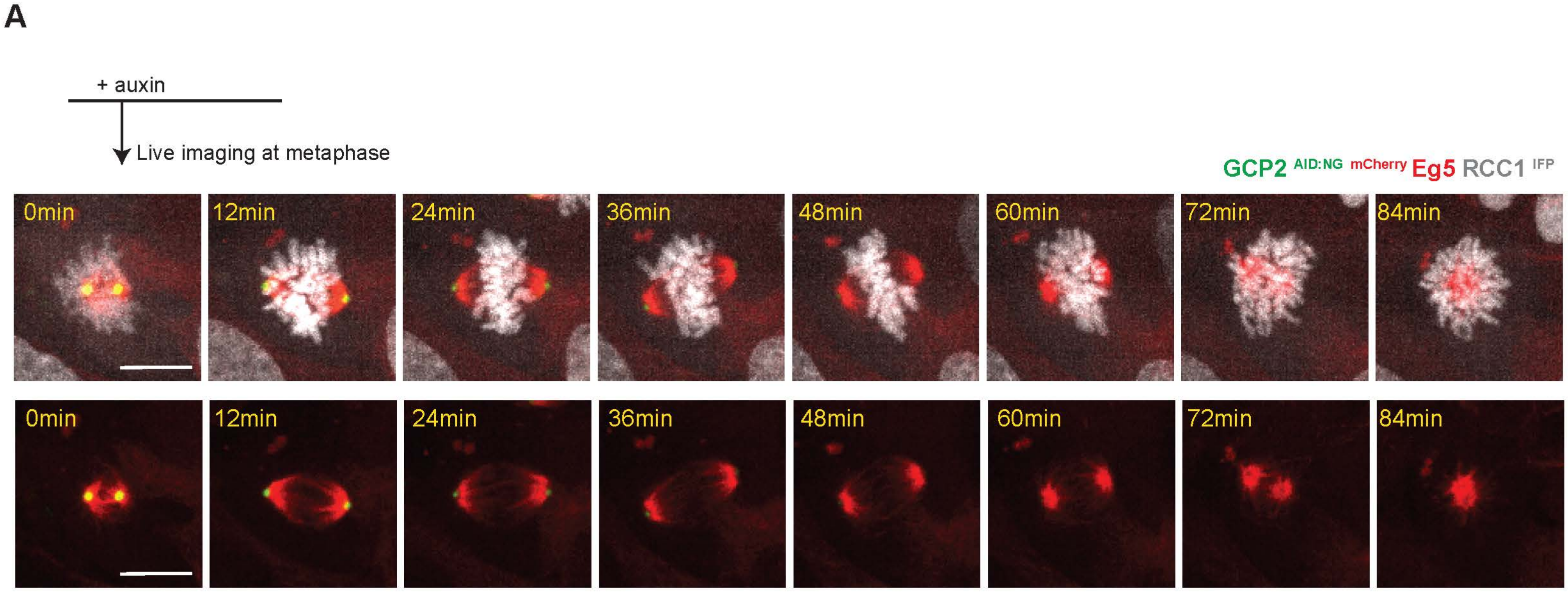

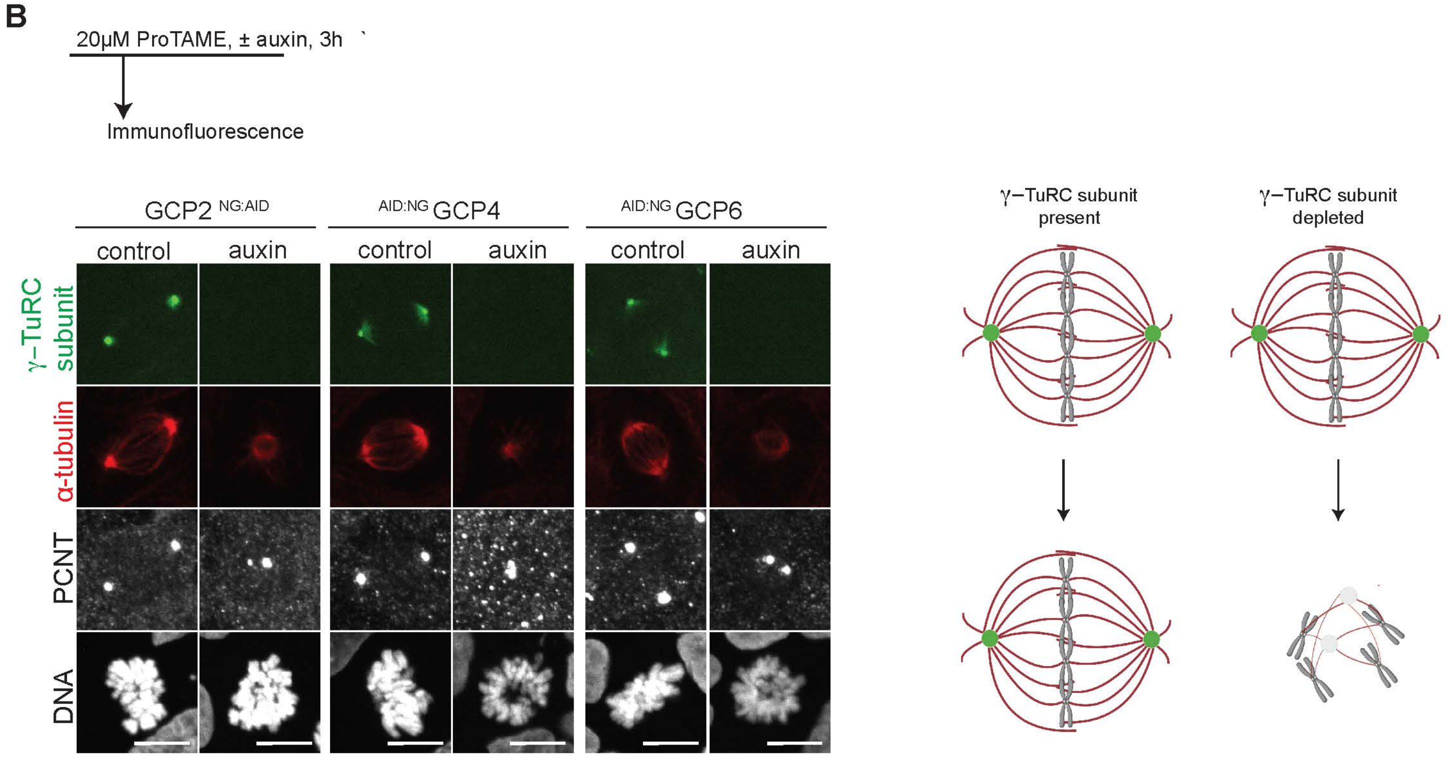

Supplementary Figure 5

bioRxiv preprint doi: https://doi.org/10.64898/2026.04.10.717779; this version posted April 11, 2026. The copyright holder for this preprint (which was not certified by peer review) is the author/funder. This article is a US Government work. It is not subject to copyright under 17 USC 105 and is also made available for use under a CC0 license.

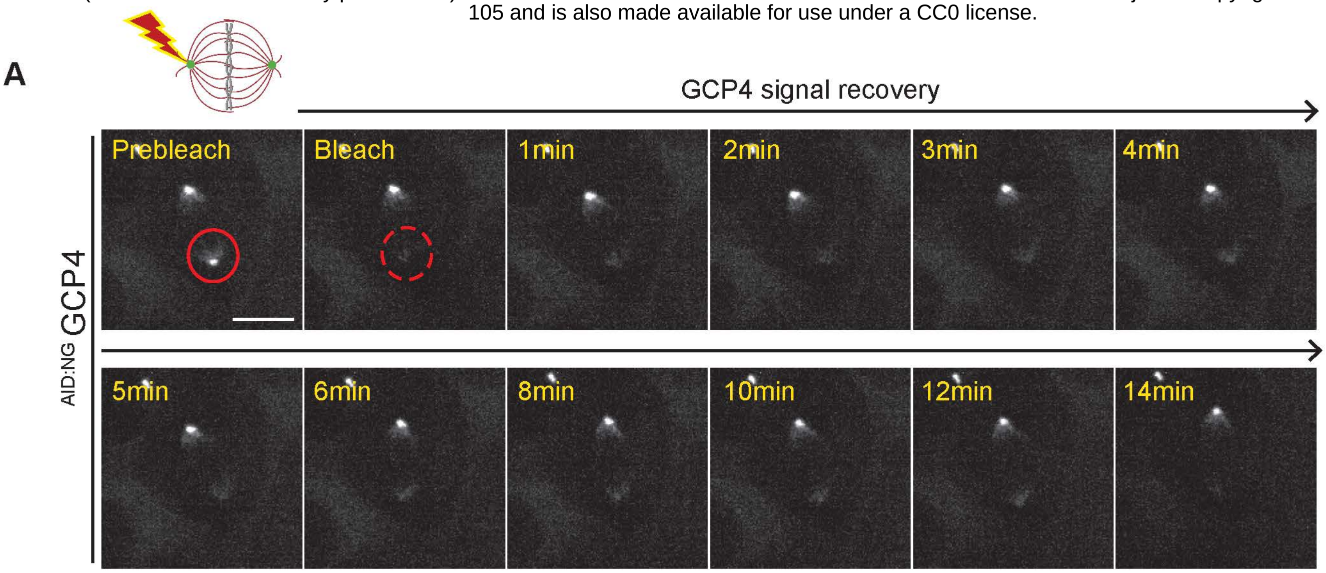

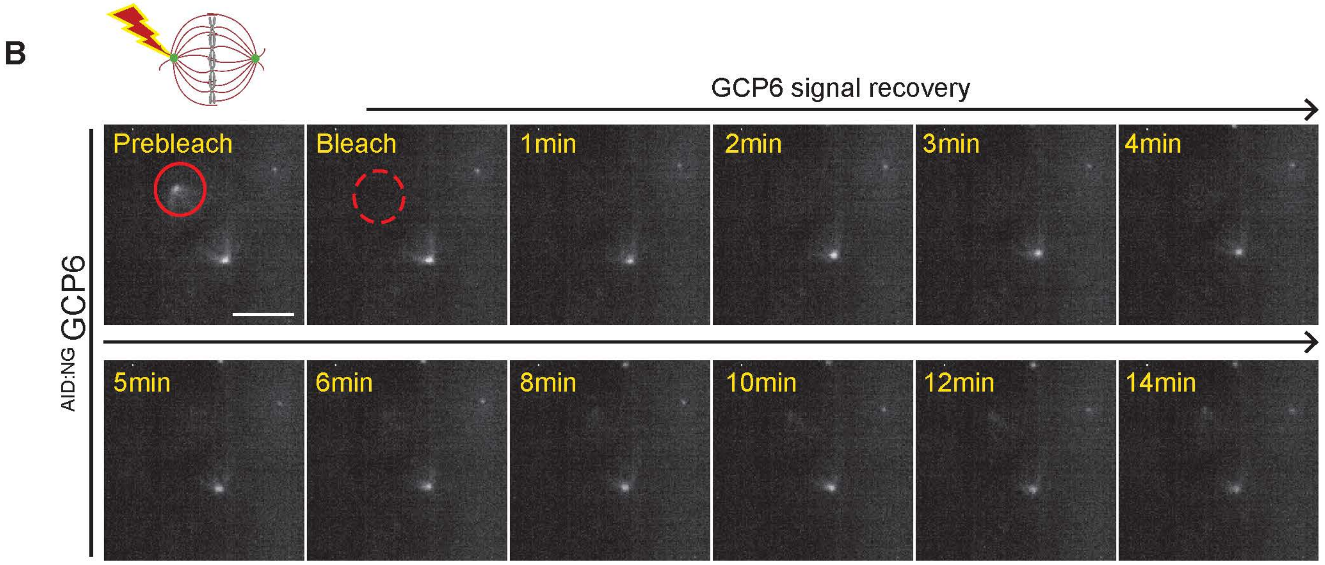

Supplementary Figure 6

bioRxiv preprint doi: https://doi.org/10.64898/2026.04.10.717779; this version posted April 11, 2026. The copyright holder for this preprint (which was not certified by peer review) is the author/funder. This article is a US Government work. It is not subject to copyright under 17 USC 105 and is also made available for use under a CC0 license.

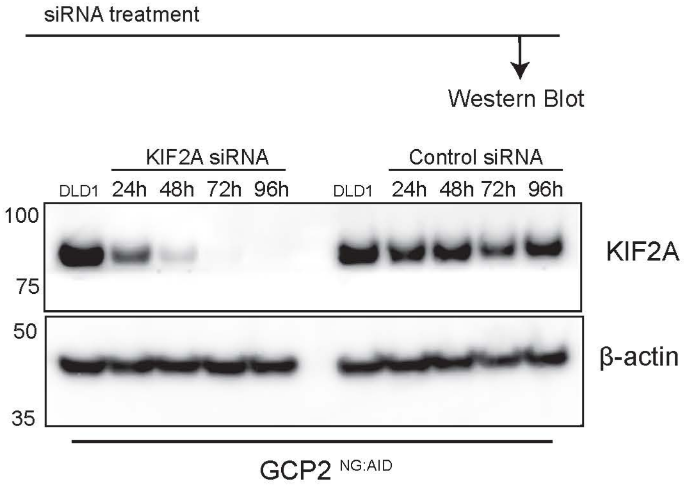

Supplementary Figure 7
